# Tuberculosis after Preventive Therapy in Persons Living with HIV Initiating Antiretroviral Therapy (Response)

**DOI:** 10.3201/eid3208.260828

**Published:** 2026-08

**Authors:** Lindsay Templin, Yagna Varajidas, Durval Respeito, Pereira Zindoga, Debora Weiss, Alexandre Nguimfack, Eudoxia Filipe, Arlete Mahumane, Maria Inês Tomo de Deus, José Mizela, Sonia Chilundo, Bruno Simoes, Aleny Couto, Ishani Pathmanathan, Emilio Dirlikov, Benedita José

**Affiliations:** US Centers for Disease Control and Prevention, Maputo, Mozambique (L. Templin, Y. Varajidas, D. Respeito, A. Nguimfack, A. Mahumane, M. Tomo de Deus, J. Mizela, S. Chilundo, I. Pathmanathan, E. Dirlikov); US Agency for International Development, Maputo (P. Zindoga); Ministry of Health Mozambique, Maputo (E. Filipe, B. Simoes, A. Couto, B. José); US Centers for Disease Control and Prevention, Atlanta, Georgia, USA (D. Weiss)

**Keywords:** Tuberculosis and other mycobacteria, HIV/AIDS and other retroviruses, bacteria, viruses, tuberculosis preventive therapy, antiretroviral therapy, Mozambique

**In Response:** We appreciate Dr. Chen’s thoughtful comments (*1*) regarding our dispatch article (*2*). In that analysis, we used routinely collected programmatic data to describe the risk of developing tuberculosis (TB) after TB preventive therapy (TPT) among persons living with HIV initiating antiretroviral therapy (ART) and did not aim to estimate a causal effect between TPT and TB. However, as Chen notes, comparisons on the basis of TPT completion are susceptible to immortal time bias and might overestimate the association between TPT exposure and TB incidence. That bias is a common gap in published literature on TB after TPT (*3*,*4*).

To address that concern, we re-analyzed the data by treating TPT exposure as a time-varying variable, allowing person-time to be appropriately classified across not started, incomplete, and complete exposure states during follow-up. In that revised analysis, adjusted incidence rate ratios (IRRs) were somewhat smaller but remained statistically significant and followed the same pattern as the previous analysis. Compared with periods following TPT completion, TB incidence was higher during incomplete TPT (adjusted IRR 1.4, 95% CI 1.3–1.5) and among those who did not initiate TPT (adjusted IRR 2.2, 95% CI 1.9–2.5).

The smaller effect estimates are consistent with what we would expect after correcting for this bias, and the fact that the overall pattern held reinforces confidence in the original findings. Our findings highlight both the value of routine programmatic data for understanding real-world TB risk and the importance of appropriate analytic approaches when interpreting such data.

